# Factors associated to quality of life of orthodontists graduated from a public university (1993-2016): A mixed-methods approach

**DOI:** 10.1590/2177-6709.25.5.23.e1-12.onl

**Published:** 2020

**Authors:** Natalia Muñoz-Pino, Anderson E. Tibaná-Guisao, Johnatan D. Cardona-Hincapié, Alejandro Hurtado-Aristizábal, Andrés A. Agudelo-Suárez

**Affiliations:** 1 Universidad de Antioquia, Facultad de Odontología (Medellín, Colombia).

**Keywords:** Quality of life, Orthodontists, Health surveys, Dental research, Qualitative research

## Abstract

**Introduction:**

For dental professionals, including orthodontists, Quality of life (QOL) is a topic of growing concern and could be determined by objective and subjective complex factors.

**Objective:**

This study analyzed the factors that influence the QOL of orthodontists graduated between 1993 and 2016 of a public university (Medellín, Colombia).

**Methods:**

A mixed-methods study was conducted (cross-sectional survey; 88 participants; 3 focus groups, 21 participants). Quantitative analysis: the research included sociodemographic, labor and health characteristics as independent variables and the WHOQOL-BREF questionnaire as main outcome for QOL. Frequencies were calculated and the association between QOL and independent variables was estimated by bivariate analysis (Chi square tests) and a linear multivariate regression. Qualitative analysis: Narrative content analysis according to thematic categories. Mixed methods: a conceptual framework for QOL using the triangulation was developed.

**Results:**

All the scores surpassed 55 points on the 4 domains of WHOQOL-BREF. A lower value was found in the physical dimension (57.1±10.7) and a greater value in the psychological dimension (70.8±8.3). The variables associated positively to QOL were permanent contract, teaching/research activities, monthly income, resting days per week and sex. Factors associated negatively were low social support, mental health and rent housing. Discourses of participants allowed to identify the concept of QOL and the contextual and social determinants and satisfiers.

**Conclusion:**

QOL of orthodontists is influenced by sociodemographic, employment, working and health factors. Therefore, QOL is a multidimensional concept that recognizes the political and socio-economic context and personal and professional experiences.

## RESUMO

**Introdução:**

Entre os profissionais da área odontológica, incluindo os ortodontistas, a Qualidade de Vida (QOL) é um tópico de interesse crescente, podendo ser determinada por meio de complexos fatores objetivos e subjetivos.

**Objetivo:**

O presente estudo analisou fatores que influenciam a QOL dos ortodontistas graduados entre 1993 e 2016 em uma universidade pública (Medelín, Colômbia).

**Método:**

Foi conduzido um estudo de metodologia mista (pesquisa transversal; 88 participantes; 3 grupos focais, 21 participantes). Análise quantitativa: foram incluídas características sociodemográficas, ocupacionais e de saúde como variáveis independentes e o questionário WHOQOL-BREF como resultado principal para QOL. As frequências foram calculadas e a associação entre QOL e as variáveis independentes foram estimadas por uma análise bivariada (teste qui-quadrado) e uma regressão linear multivariada. Análise qualitativa: análise do conteúdo narrativo de acordo com as categorias temáticas. Metodologia mista: foi desenvolvida uma estrutura conceitual para QOL utilizando a triangulação.

**Resultados:**

Todas as pontuações ultrapassaram 55 pontos nos 4 domínios do WHOQOL-BREF. Um valor mais baixo foi encontrado na dimensão física (57,1±10,7) e um valor mais alto, na dimensão psicológica (70,8±8,3). As variáveis associadas positivamente à QOL foram as seguintes: contrato permanente, atividades ensino/pesquisa, renda mensal, dias de descanso por semana e sexo. Os fatores associados negativamente foram baixo suporte social, saúde mental e morar de aluguel. O discurso dos participantes permitiu a identificação do conceito de QOL, dos determinantes contextuais e sociais, e os fatores motivacionais.

**Conclusão:**

A QOL dos ortodontistas é influenciada por fatores sociodemográficos, ocupacionais, ligados ao trabalho e à saúde. Dessa maneira, a QOL se apresenta como um conceito multidimensional, que reconhece o contexto político e socioeconômico, assim como as experiências pessoais e profissionais.

## INTRODUCTION

Quality of life (QOL) is a broad concept that is affected by objective and subjective factors such as physical health, psychological status, level of independence, social support, personal beliefs and the environment.^1,2^ The multidimensional connotation of QOL has been studied in diverse population groups and is recognized as a dynamic process, because of the factors that can influence over the time.^2-5^ According to the United Nations Development Programme (UNDP) and its Human Development Report (2018), Colombia has a high Human Development Index of 0.777 (on a scale of 0 to 1), ranking 90th worldwide.^6^ Specifically in Medellin (the second largest city in the country), the municipal government carried out the multidimensional index of living conditions, and the perception of QOL in the region is 0.653 (values ​​close to 1 indicate a better situation).^7^

For dental professionals, QOL is currently a topic of growing concern.^8-10^ Dentists at working place are under a constant pressure that demands skills, physical energy and concentration. They are exposed to a number of occupational diseases, and they are vulnerable to multiple hazards: ergonomic, physical, chemical, biological, and psychosocial.^10^ ^,^ ^11^ In addition, they have a lot of necessities for education and training in order to offer update knowledge and technology to keep up with patients’ increasing demand. ^8-10^

The neoliberal economic model adopted in the country has brought changes in the practice of autonomous health professionals, including general dentistry and its specialties (such as orthodontics).^8,12^ Therefore, an oversupply of general dentists and specialists is observed in some regions of the country, and this situation impacts in the precarious working conditions, having multiple jobs, long working days, and inter-municipal transfers for working in different dental offices.^8,12^ In addition, the city of Medellín (and its metropolitan area) has four dentistry schools (three of them are private) and three universities have orthodontics specialization programs (one of them is public). These aspects can affect the QOL of professionals and consequently the ability to provide high quality dental care.^8^

International research about the QOL in dentists have been found, but not in a broad way. For instance, one study conducted in Brazil^5^ identified a low QOL related to physical and psychological health. Other studies conducted in India^9^ and United Arab Emirates^10^ analyzed the sociodemographic and labor characteristics that influence QOL in different settings. No empirical studies have been identified in Colombia that measured QOL in dentists, but studies related to the work profile, living conditions, work-life balance and job satisfaction in these professionals are found.^12-14^ For that purpose, new methodological approaches are requested to address broadly the social determinants affecting QOL in orthodontists. In this case, mixed methods are increasingly being used in research studies on multifunctional oral health issues.^15^ The knowledge of the social reality of these professionals permits generating strategies and political actions to improve health. 

Accordingly, this study aims to analyze the factors that influence the QOL of the orthodontists graduated between 1993 and 2016 from a public university (Colombia). 

## METHODS

### Ethics

Participation in the study was voluntary. All respondents gave written informed consent to participate in the study, and confidentiality was guaranteed throughout the research process in accordance with Colombian regulations (Resolution no. 008430/1993- Ministry of Health and Social Protection). The Ethical Committee of the Faculty of Dentistry at University of Antioquia approved the study (07-2017).

### Design

A mixed-methods study was conducted by means of a sequential explanatory design,^15^ which means that qualitative tools were used to explain what was observed in the first quantitative phase. Fieldwork was carried out between June 2017 and July 2018. All the components are referenced below.

### Quantitative sub-study

A cross-sectional survey was applied for orthodontists graduated from the Faculty of Dentistry of the University of Antioquia (Medellín, Colombia). Data were provided by the *Asociación de Ortodoncistas de la Universidad de Antioquia* (Association of Orthodontists of the University of Antioquia). The final sample was 88 (response rate: 68%), considering a voluntary participation. For the correct filling of the surveys, the research team attended several meetings scheduled by the Association. The questionnaire was self-administered, but advice was given if needed and were later delivered to the researchers during all the events (questionnaire available upon request). All surveys were anonymous and confidential. A pilot study was carried out in a sample of 10 dentists in order to improve intelligibility and to assess time to completion and internal consistency.

The main outcome was the Health-Related Quality of Life (HRQOL), as measured by the WHOQOL-BREF.^16,17^ This is a generic questionnaire to measure QOL, created by the Study Group on Quality of Life of the WHO, and comprises 26 items distributed on four broad domains: physical health, psychological health, social relationships, and environment. Domain scores are scaled in a positive direction from 1 to 5 (i.e., higher scores denote higher quality of life). All the items give a raw score, which is transformed to a 0-100 scale, according to the recommendations of the Study Group. This questionnaire has been validated and is available in 19 languages, including Spanish.^16^ ^,^ ^17^

Explanatory variables were included: employment conditions, socio-demographics, mental health (measured with the 12-item version of the General Health Questionnaire GHQ-12, responses were rated and summed, and individuals with a score of 3 or higher were classified as having poor mental health).^18^ The Duke-UNC-11 questionnaire was used to measure social support. This instrument containing 11 items evaluates perceived functional or qualitative social support. Each item is scored on a frequency rating from 1 (*“Much less than I would like”*) to 5 (*“As much as I would like”*). The score was calculated by adding up the responses to each item, with a higher score denoting greater social support. The cut-off point for low levels of social support is the 15^th^ percentile, corresponding to a score of 32.^19^

A descriptive analysis was carried out for all variables. The Kolmogorov-Smirnov test was used for verifying normality distribution in the main outcome. A bivariate analysis was conducted for the scores of the domains of HRQOL with qualitative explanatory variables, and tests of statistical significance were carried out to observe differences among variables, according to their nature (Mann-Whitney U test, for dichotomous variables; Kruskal-Wallis test, for polychotomous variables; and the Spearman correlation, for quantitative variables). A linear multivariate regression analysis was carried out in order to evaluate the simultaneous and reciprocal effect of the explanatory variables on each of the dimensions of WHOQOL-BREF and to identify possible predictors of their scores. Belonging was determined by evaluating the compliance with the assumptions of linearity, non-collinearity and normality, constant variance and correlation of residuals. All analyses used a level of statistical significance of <0.05. SPSS software version 22.0 (IBM^®^) was used to carry out all of the analyses.

### Qualitative sub-study

A qualitative approach (focused ethnographic perspective) was conducted by means of three focus group discussions (FGD) and participated 21 orthodontists that previously completed the survey (selected for convenience). The research team produced a guide for use in the FGDs that indicated a series of topics to be discussed among participants (according to quantitative survey).The FGD were conducted by two members of the research team. FGDs lasted between 60 and 90 min, and were digitally recorded and transcribed literally. FGDs were performed until data saturation was reached, meaning that no new information emerged.

Narrative content analysis was conducted, identifying significant pieces of text and trends of information found in the participants’ discourse. Data analysis were conducted by three of the authors, who examined and compared their analyses. Transcribed data were imported into the qualitative analysis software Atlas. Ti 8.0 and the final analysis was supervised by one of the research team. The text fragments were labeled in 126 codes and then grouped into three categories. 

### The methods integration approach

The integration of both sub-studies was carried out by means of triangulation.^13^ A conceptual framework was formulated, identifying the factors influencing QOL in several levels, according to individuals’ opinions and social factors related to the particularities of this orthodontists’ group in Colombia. 

## RESULTS

### Sociodemographic, labor, health and QOL profile of participants

General profile for the study sample is provided in a supplementary table. Participated 88 orthodontists (52% females), with an average age of 42±7 years. The median scores for QOL according to the WHOQOL-BREF were over 50, with the best score in the psychological dimension (Me= 71; IQR= 8) and the worst score in the physical dimension (Me= 57; IQR= 11). 66% practice sports; 14% perceived poor general health, 23% poor mental health and the social support is considered as low in 6% of cases. 

**Supplementary Table:** Sociodemographic, labour, health and quality of life variables in the study sample. Medellín, 2018 (n=88).

Variables	n (%)
Sociodemographic	
*Sex*	
Males	42 (47.7)
Females	46 (52.3)
*Age* ^*a*^	
Mean (± SD)	42.2 (7.1)
*Marital status*	
Single	26 (29.5)
Married / Cohabited	55 (62.5)
Separate	7 (8.0)
*Socioeconomic status*	
Middle	24 (27.6)
High	63 (72.4)
*Housing*	
Own	69 (78,4)
Rented	11 (12.5)
Other	8 (9.1)
*Vehicle*	
Yes	80 (90.9)
No	8 (9.1)
*Type of family* ^*c*^	
Nuclear	62 (72.1)
Extended	5 (5.8)
Single-parent	3 (3.5)
Assembled	1 (1.2)
Live alone	15 (17.0)
*Number of people in charge* ^*a*^	
Median (IQR)	1.0 (2.0)
Labour conditions	
*Laboractivity* ^*b*^	
Teaching/research	27 (30.7)
Clinical assistance	88 (100.0)
Administrative	9 (10.2)
Other	2 (2.3)
*Written contract*	
Yes	57 (64.8)
No	31 (35.2)
*Presence of several contracts*	
Yes	51 (58.0)
No	37 (42.0)
*Type of contract* ^*b*^	
Permanent	9 (10.2)
Temporal	16 (18.2)
Independent	62 (70.5)
Provision of services	52 (59.1)
Percentage rent	53 (59.1)
*Percentage rent value* ^*a*^	
Median (IQR)	60.0 (20.0)
*Working hours per week* ^*a*^	
Mean (± SD)	38.9 (12.0)
*Resting days per week* ^*a*^	
Median (IQR)	2.0 (1.0)
Variables	n (%)
*Working geographical regions* ^*b*^	
Metropolitan area	78 (88.6)
Intermunicipal	63 (71.6)
Interstate	2 (2.3)
*Average income (Colombian peso)* ^*d*^	
3,000.001 - 4,000.000	5 (5.7)
> 4,000.001 - 5,000.000	9 (10.2)
> 5,000.001 - < 7,000.000	16 (18.2)
> 7,000.000	58 (65.9)
*Number of work places as an orthodontist* ^*a*^	
Median (IQR)	4.0 (3.0)
*Does your current salary allow you to cover your basic needs, and those of the people who depend on you?*	
Yes	80 (90.9)
No	8 (9.1)
*Does your current salary allow you to cover* *unforeseen important expenses?*	
Yes	66 (75.0)
No	22 (25.0)
*Training and unformal education* ^*b*^	
Diploma courses	33 (37.5)
Short courses	78 (88.6)
Congress	86 (97.7)
Conferences	67 (76.1)
Other	4 (4.5)
*Annual frequency of participation in events of training* *and unformal education* ^*a*^	
Median (IQR)	3.0 (3.0)
Quality of life	
*Physical* ^*a*^	
Median (IQR)	57.1 (10.7)
*Psychological* ^*a*^	
Median (IQR)	70.8 (8.3)
*Social* ^*a*^	
Median (IQR)	66.6 (25.0)
*Environment* ^*a*^	
Median (IQR)	68.7 (18.7)
Health	
*Sport practice*	
Yes	58 (66.0)
No	30 (34.0)
*Body Mass Index (BMI)* ^*a*^	
Mean (± SD)	24.1 (3.4)
*Self-rated health* ^*c*^	
Good	71 (85,5)
Poor	12 (14.4)
*Social support (Duke-UNC-11)*	
Normal	83 (94.3)
Low	5 (5.7)
*Mental health (GHQ-12)*	
Good	68 (77.3)
Poor	20 (22.7)

### Sociodemographic, labor and health aspects associated with QOL in their dimensions

Table 1 shows the bivariate correlations between the WHOQOL-BREF dimensions of QOL and the quantitative variables in the study sample. A negative statistically significant correlations was found in case of years of graduation as orthodontist and the social dimension (the score of QOL in this dimension is lower in more recent graduated orthodontist). For the environment dimension of QOL, negative statistically significant correlations for the number of family people in charge and working hours per week (the score of QOL in this dimension is lower in those participants with a greater number of people in charge and those having more working hours per week). A positive statistically significant correlations were found for the variable resting hours per week and the environment dimension (the QOL is higher in those participants with more resting days per week).

**Table 1:** Correlations between the WHOQOL-BREF dimensions of quality of life and the sociodemographic, labor and health variables in the study sample. Medellín, 2018 (n= 88).

Variables	WHOQOL-BREF dimensions			
	Physical	Psychological	Social	Environment
Age	0.2	-0.02	0.1	-0.1
Years of graduation as orthodontist	-0.02	-0.04	-0.3*	-0.14
Number of people in charge (family)	-0.12	-0.1	-0.1	-0.3*
Working hours per week	-0.1	-0.02	-0.04	-0.3*
Resting days per week	0.04	0.06	0.2	0.3**
Number of jobs	-0.13	-0.1	-0.05	-0.14
Body Mass Index	-0.07	-0.12	-0.03	-0.2

Spearman’s rank correlation coefficient. * p-value < 0.05 and > a 0.01. ** p-value < 0.01 and > a 0.001*. *** p-value <0.001.

Table 2 shows the bivariate comparison between the WHOQOL-BREF dimensions of QOL and the qualitative variables in the study. Statistically significant differences in the median scores were found for the physical dimension and the variables: teaching/research and other labors, temporal contracts, mental health. For the psychological dimension, statistically significant differences in the median scores were found in case of the variables: teaching/research labors, temporal contracts, self-rated health and mental health. For social dimension of QOL, statistically significant differences in the median scores were observed for the variables: social support and mental health. Finally, for the environment dimension, statistically significant differences in the median scores were observed according to the variables: sex, housing, social support, self-rated health and mental health. 

**Table 2:** Bivariate comparison of the WHOQOL-BREF dimensions of quality of life and the qualitative variables in the study sample. Medellín, 2018 (n= 88).

Variables	WHOQOL-BREF dimensions											
	Physical			Psychological			Social			Environment		
	Me	IQR	p-value	Me	IQR	p-value	Me	IQR	p-value	Me	IQR	p-value
Sex												
Males	55.4	10.7	0.86	70.8	12.5	0.88	66.7	16.7	0.55	68.8	15.6	0.019*
Females	57.1	14.3		70.8	8.3		66.7	25.0		71.9	15.6	
Marital status												
Single	55.4	14.3	0.69	70.8	16.7	0.66	66.7	25.0	0.99	70.3	15.6	0.81
Married /Cohabitated	57.1	14.3		70.8	8.3		66.7	16.7		70.3	15.6	
Separate	53.4	10.7		66.7	12.5		66.7	25.0		68.8	10.8	
Socioeconomic status												
Middle	57.1	10.7	0.62	70.8	12.5	0.99	66.7	20.8	0.73	70.3	20.3	0,45
High	53.6	10.7		70.8	8.3		66.7	25.0		68.8	15.6	
Housing												
Own	57.1	14.3	0.06	70.8	8.3	0,07	66.7	25.0	0.25	68.8	18.8	0.031*
Rented	50.0	14.3		66.7	8.3		66.7	16.7		59.4	18.8	
Other	57.1	5.4		77.1	10.4		79.2	16.7		73.4	10.9	
Sport practice												
Yes	55.4	14.3	0.94	70.8	8.3	0.96	66.7	25.0	0.64	71.9	15.6	0.082
No	57.1	14.3		70.8	8.3		66.7	16.7		67.2	18.8	
Labor activity - Teaching/research												
No	53.6	10.7	0.019*	70.8	12.5	0.005**	66.7	16.7	0.44	68.8	15.6	0.51
Yes	60.7	14.3		75.0	12.5		75.0	25.0		68.8	18.8	
Clinical assistance												
No	NC	NC	----	NC	NC	----	NC	NC	----	NC	NC	----
Yes	57.4	12.5		70.8	8.3		66.7	25.0		68.8	17.2	
Administrative												
No	53.6	10.7	0.22	70.8	8.3	0.22	66.7	25.0	0.44	68.8	18.8	0.23
Yes	57.1	7.2		75.0	12.5		75.0	16.7		71.9	3.1	
Other												
No	53.6	10.7	0.012*	70.8	8.3	0.32	66.7	16,7	0.81	68.8	18.8	0.49
Yes	64.3	7.1		70.8	12.5		75.0	33.3		68.8	15.6	
Presence of several contracts												
Yes	55.4	14.3	0.67	70.8	8.3	0.43	66.7	25.0	0.35	68.8	15.6	0.5
No	57.1	10.7		68.8	12.5		66.7	16.7		68.8	15.6	
Written contract												
Yes	57.1	14.3	0.25	70.8	8.3	0.27	75.0	25.0	0.16	68.8	15.6	0.34
No	53.6	10.7		70.8	16.7		66.7	16.7		68.8	15.6	
Type of contract - Independent												
No	57.1	10.7	0.76	70.8	8.3	0.996	66.7	8.3	0.9	71.9	15.6	0.62
Yes	53.6	14.3		70.8	8.3		66.7	25.0		68.8	15.6	
Provision of services												
No	57.1	12.5	0.16	70.8	10.4	0.31	66.7	16.7	0.55	68.8	20.3	0.65
Yes	53.6	10.7		70.8	9.2		66.7	25.0		68.8	14.1	
Percentage rent												
No	57.1	12.5	0,097	68.8	12.5	0.6	70.8	20.8	0.64	70.3	15.6	0.37
Yes	53.6	12.5		70.8	8.3		66.7	25.0		68.8	15.6	
Temporal												
No	53.6	10.7	0.037*	70.8	12.5	0.019*	66.7	20.8	0.36	68.8	15.6	0.396
Yes	60.7	12.5		75.0	12.5		75.0	20.8		68.8	20.3	
Variables	WHOQOL-BREF dimensions											
	Physical			Psychological			Social			Environment		
	Me	IQR	p-value	Me	IQR	p-value	Me	IQR	p-value	Me	IQR	p-value
Average income (Colombian peso)												
3.000.001- 4.000.000	57.1	3.6	0.31	75.0	0.0	0.16	75.0	25.0	0.71	71.9	6.3	0.63
>4.000.001- 5.000.000	57.1	14.3		70.8	0.0		66.7	16.7		62.5	12.5	
>5.000.001-< 7.000.000	58.9	19.6		70.8	12.5		75.0	33.3		67.2	21.9	
> 7.000.000	53.6	10.7		68.8	14.6		66.7	16.7		68.8	15.6	
Self-rated health												
Good	51.8	14.3	0.25	66.7	10.4	0,05*	58.3	12.5	0,16	60.9	14.1	0,013*
Poor	57.1	14.3		70.8	8.3		75.0	25.0		71.9	15.6	
Social support (Duke-UNC-11)												
Normal	57.1	14.3	0.088	70.8	8.3	0.277	66.7	25.0	0.005**	68.8	15.6	0.02*
Low	42.9	14.3		66.7	8.3		33.3	0.0		56.3	0.0	
Mental health (GHQ-12)												
Good	57.1	14.3	0.004**	70.8	8.3	<0.001***	75.0	16.7	0.003**	71.9	14.1	0.001**
Poor	50.0	16.1		64.6	10.4		58.3	20.8		60.9	12.5	

Mann Whitney U test for dichotomous variables, Kruskal-Wallis test for polychotomous variables.

* p-value < 0.05 and > a 0.01 ** p-value < 0.01 and > a 0.001* *** p-value <0.001.

### Potential predictors of QOL

According to the multivariate linear regression models (Table 3), poor mental health was a negative predictor variable in physical, psychological, and environment dimensions (having poor mental health is associated with a low QOL). To have a rent house was a negative predictor in case of physical and environment dimensions. To work in labors related to teaching/research was a positive predictor variable for QOL in the psychological and environment dimensions. Social support (low) was a negative predictor variable for the psychological dimension of QOL. Finally, to have resting days per week, sex (to be a woman) and monthly income were positive predictors for QOL for the environment dimension. The independent variables described explained between 16 percent and 40 percent of the scores obtained for the dimensions. Mental health had the most statistical weight for almost all dimensions of QOL, with a standardized regression coefficient between -0.4 and -0.5 (Table 3).

**Table 3:** Lineal regression models for the scores of the WHOQOL-BREF dimensions of QOL in the study sample. Medellín, 2018 (n= 88).

WHOQOL-BREF dimensions	Variables included in the lineal regression model	Non-standardized Regression Coefficient	Standardized Regression Coefficient	Determination Coefficient (%)
Physical	Mental Health (Poor)	-8.5***	-0.4	21.0
	Rent housing	-8.9**	-0.3	
	Permanent contract	7.6*	0.2	
Psychological	Mental Health (Poor)	-8.2***	-0.4	25.0
	Labour activity: Teaching/researcher	6.6***	0.4	
Social	Social support (Low)	-30.0***	-0.4	16.0
Environment	Mental Health (Poor)	-13.28 ***	-0.5	41.0
	Rent housing	-11.33**	-0.3	
	Sex (Females)	7.09**	0.3	
	Resting days per week	3.96**	0.3	
	Labour activity: Teaching/researcher	5.8*	0.2	
	Monthly income	5.1*	0.2	

* p-value < 0.05 and > a 0.01. ** p-value < 0.01 and > a 0.001*. *** p-value <0.001.

### Participants’ opinions and perspectives

Participants consider QOL a multidimensional and dynamic element where subjective elements of the personal and professional experience intervene as factors of the social, cultural and economic context of the country that enable the exercise of the profession and obtainment of personal, familiar and work gain (Table 4, 1a, 1b). Similarly, QOL implies the satisfaction of needs and the compliance with different expectations considered important for daily enjoyment (Table 4, 1c). 

**Table 4:** Verbatim focus discussions groups (FDG) extracts form participants’ discourses (3 FDG, n = 21).

Categories	Verbatim extracts from participants
1. Concept of quality of life (QOL)	a) *“If someone has professional success, they have all the conditions to have a better quality of life. Professional success does not necessarily mean working from 7 to 7, as I will probably be successful but won’t have a good quality of life. Quality of life encompasses many things”* (FDG 2)
	b) (...) *“I think that quality of life is like a state of well-being, right? It needs to be differentiated from individual quality of life and that of, say, society or a community or a group of people or a society as the individual has aspirations, desires, anxieties as they say. As long as that is given back, I would say they have a good quality of life. At a social level, there has to be some standards that say which is the quality of life for that society and so each individual will have a way to be integrated into that society in terms of quality of life and in regards to their dreams of quality of life”* (FDG 3)
	c) *“I consider quality of life is doing everything that makes us happy and having the conditions that allow for it; staying at home if that makes me happy but doing it because I can afford it, or play sports, or travel or study but being at ease with being able to do things and do and achieve what allows me to do it”* (FDG 1)
2. Negative and positive determinants/conditionings of Quality of Life (QOL)	a) *“I think that anyway that result can be associated to that even though we have greater mental demand -for all we have said, it is not physically that demanding whereas when you work as an orthodontist the physical demand is greater while the mental one is, say, may not be so high. As teachers, anyway, I think we all choose to be here since it gives us a level of satisfaction as we are not expecting further retribution other than being happy with what we do. I also share that being professors demands more time outside the work place”* (FDG 3)
	b) *“I would love to have social benefits and saying for once in my life that I will have a smooth December. For me December is torture because it means paying all December-related and January expenses without having the money because people chooses not to go to treatments and so I am like “come let’s work until Christmas”*( FDG 1)
	c) *”Financially, I don’t think being an orthodontist is profitable these days. To have high income you need to work very hard and long hours, to achieve that balance”* (FDG 3)
	d) (...) *“we have spoken about muscle-skeletal disorders. We are very exposed to them. Obviously as more patients, greater the risk of having some health issue that will affect work performance and quality of life”* ( FDG 3)
	e) (...) *“It makes me laugh because one day we met, all the female alumni from Universidad de Antioquia, mostly from orthodontics, and they began to talk… all went to the same therapist! They had to see a therapist because it was hard to cope with day-by-day activities and stress. All, because of their personality type, began a zero-stress program… not stressing over things you cannot control as traffic jams. As they say around here a lot, light one and relax as you arrive, and all that”* (FDG 1)
	f) *”I think that for myself, it is just a mystery because when the control body announces a visit everyone is stressed, at least from two months before. For me… I don’t see any more than external pressure; the pressure on the professional”* (FDG 2)
	g) *”One thing we have not covered is that the health system is also affecting us, particularly orthodontists. For example, a dentist thinking about opening a practice. From 150 graduating, 10 opened their private practice and most are thinking about finding work. That is also seen in specialists when other entities are beginning to cover specializations of dentistry, they also will absorb the demand and we will obviously see the amount of patients decrease from the private practice. That will impact the level of income because health service providers are absorbing it”* (FDG 3)
	h) *“I agree with that payment is not the same but satisfactions are greater since we work better each time. Each time we have more experience, because each time we manage people better”* (FDG 2)
3. Proposals and strategies for improving QOL in orthodontists	a) *“Here is a point I find very important, what he says. Our programs, as I understand, in other countries students up to their 20’s, or the first third of their lives, are taught economics, administration and we are ignorant in those fields. We charge for what we observe but we do not know why. Besides we don’t know how to invest what we make. They are prepared for that so when they go out to the productive life they already know what to do with their money. We don’t have that clear”* (FDG 2)
	b) *“We talked about it once, as (name of the person) said, doctors saw a way to organize themselves, We do not know how to be organized towards the same goal. If we all fought in the same direction this would be different”.* (FDG 2)
	c) (...) *“I had to make a bunch of changes in work-hours, pause, join the gym, strengthen my back, abdomen… I even stop exercising for over a month and the body claims it. I begin work and the back feels warm and I know I have to start back exercises”.* (FDG 1)

Another category is related with conditionals/determinants of QOL, which operates in positive or negative ways. Employment and work conditions of orthodontists differ among interviewees depending on the type of contract and activity (Table 4, 2a). When analyzing the specific working conditions of orthodontists who do clinical practice, they mention, for example, absence of benefits, long working hours, and emotional burden (Table 4, 2b). They also report a detriment to working conditions in terms of income (Table 4, 2c), impacting the physical and mental health situation (Table 4, 2d, 2e).

The exercise of the profession is marked by the current legislation on Social Security, and by elements of the Enforced Health Quality Assurance System that, in the opinion of the participants, has permeated in some difficulties to work in their private offices due to operating requirements (Table 4, 2f, 2g). High market competition is referred to by orthodontists graduated from other universities in the city and the country and from general dentists who perform low cost orthodontics (Table 4, 2g).

Participants mentioned some satisfaction of QOL as, for example, saving for the future, family support, teaching, support networks as, in this case, the association of orthodontists. Similarly, the positive transformation resulting from graduate schooling is highlighted as mind-opening as it gives them satisfaction as they consider their job useful to society (Table 4, 2h). 

Interviewees present a series of proposals to improve QOL. First, they mention some strategies to be implemented in graduate school: financial education, administrative aspects, enterprise creation and entrepreneurship (Table 4, 3a). The importance of associations is highlighted as a strategy to attain common objectives as orthodontist in exercise of the profession, as compared with other professions with more guild experience (Table 4, 3b). Lastly, they refer some individual strategies as, for example, time management, promotion of health and spare time (Table 4, 3c).

### Understanding individual, social and contextual factors influencing QOL in orthodontists: a conceptual explicative framework

**Figure 1:**
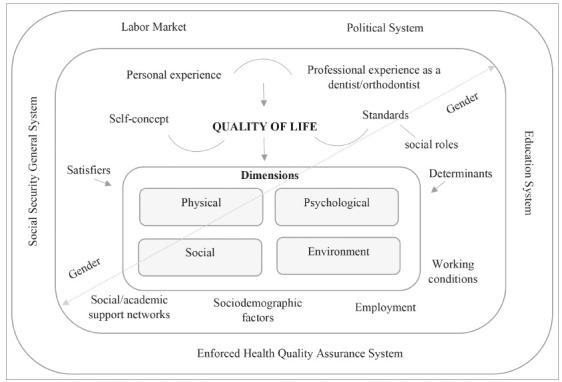
Conceptual explanatory framework for QOL in orthodontists according to quantitative and qualitative findings.

Figure 1 shows the conceptual framework to understand several factors related to QOL from the participants’ perspective. QOL appears as a multifactorial construct based on personal and professional subjective experiences, in sociodemographic, labor and social characteristics and all influenced for the political, social and economic context of the country. 

## DISCUSSION

This study analyzed the influence of socio-demographic and job characteristics on QOL of orthodontists graduated from a public university in Medellin (Colombia). Main scores surpassed 55 points on the four domains of WHOQOL-BREF, which suggest a good quality of life of the population studied. In spite of that, a lower value is found in the physical dimension and a greater value in the psychological dimension. In relation to such results, a study on dentistry specialists with QOL using the same instrument revealed a better value in the social and psychological domains and lower value in the environmental domain.^8^ Another study on dentists showed high QOL in the physical and psychological domains, and a lower value in the environmental domain.^5^ The variability in findings could be explained according to the social and economic setting of the analyzed studies. 

In spite of the heterogeneity of results, it is possible to think that variability in score in the environmental domain measured by WHOQOL-BREF, as found between the studies, is due to uneven conditions in factors related with financial resources, safety, health, social assistance and opportunities for recreational activities of each population.^10^ The finding related with lower QOL value in the physical domain, which measures categories related with energy, fatigue, pain, discomfort, sleep and rest, could be related to a degree with the effects inherent to the exercise as orthodontist, including musculoskeletal disorders, which are highly reported by dentists,^18^ thus affecting QOL and reducing work productivity. Reported risk factors at work include working in the same or in uncomfortable positions for long periods of time and seeing an excessive number of patients a day.^19^

The psychological domain presents a higher value in the group studied, which measures aspects related to body image, appearance, negative and positive feelings, self-esteem, thoughts, apprenticeship, memory and concentration. This result concurs with other on QOL of dentists in teaching hospitals, showing that becoming a specialist has a positive impact in the QOL in the psychological domain.^9^ Graduate school can undoubtedly influence the level of self-esteem and improve positive feelings in an individual.^9^ Furthermore, it was found that dentists with a postgraduate degree have higher levels of overall professional satisfaction which can positively influence QOL.^22^ ^,^ ^23^

Being an orthodontist was considered by FDG as not monotonous as it exercises thinking and integrates creativity and skill, having a positive impact on the psychological dimension. Even though there were no quantitative comparisons of QOL of orthodontists and general dentists, it came out in the FDG where specialists report an improvement in their QOL since becoming specialists. This was reported by another study where the differences in QOL and in the work satisfaction of the specialist are evident as the specialist focuses on the specialty and has the opportunity to collaborate with other in the field in different forum and associations, and financial rewards are also greater as compared with dentists.^10^

Even though historically male and female have taken different roles and responsibilities in regard to work and family education, studies have reported that the practice of female orthodontists is not significantly different from male.^24^ The present study makes evident better QOL of female in the environmental domain, despite the multiple responsibilities besides the profession. A qualitative study on female orthodontist reports that satisfaction with their personal and professional roles is related with the need to commit to and prioritize activities for the accomplishment of family-work balance, finding adaptations both on maternity and the professional roles.^25^

Among the factors related with working conditions of orthodontists, the influence of work-hours and days-off per week in the environmental domain of QOL was identified. In an international-scope study on orthodontists, it is reported an average of 30 work-hours per week,^23^ and the present study estimates an average of 39±12 work-hours per week. The equitable distribution of work and rest times accounts for the equilibrium between personal and work life mandatory for a good QOL, despite the multiple factors that may affect this balance.^26^ Dentistry and its specialties are autonomous professions hence the decision to distribute time will depend on the free choosing of the professional, conditioned by their needs and the environment.^26^ Also work dissatisfaction of orthodontists has been reported on aspects related with management of the practice and the amount of personal time, reinforcing the influence of work conditions on work satisfaction and consequently QOL.^27^ ^-^ ^30^

In this study, activities of teaching/researching positively influenced QOL in the physical and psychological dimensions. Possibly, these results are related with the lower physical demand and less time of clinical practice, which reduce exposure to risk factors and positively influence attributes of recognition, concentration and learning as aspects measured in the psychological dimensions of WHOQOL-BREF.^16,20^ In the FDG of professors of orthodontics is discussed that despite the economic retribution received from teaching, as is lower than income from clinical practice, the activity is better valued as they are allowed to communicate their knowledge, learn and stay updated. A similar study on professors of orthodontics reported multiple reasons to enter academia: desire to teach, opportunity to mentor, research, advisory and returning to the specialty.^29^

The greater predictor of QOL in the domains surveyed, except social relationships, was mental health being better the QOL score when reported as good. Working in a dental practice is known as physically and mentally demanding, which can generate chronic stress and affect the mental health of the professional.^10,31,32^ Furthermore, it has been described that professional exhaustion eventually leads to both mental and emotional burnout, and may end in a negative attitude both in the professional and personal scopes, affecting the overall health of the individual.^31^ ^,^ ^32^ From the opinions of orthodontists part in the FDG emerge ideas related with the concept of work stress as a factor affecting QOL that cannot only influence the psychological but also the physical dimensions. 

Limitations of the present study include the rate of response of participants and conformation of FDG. This may affect the results of the study as the decision to reply the survey or participate in focus groups may be related with perceptions of their quality of life and family and work conditions. Similarly, the sampling selection makes generalizing conclusions about all the population of orthodontists at regional and national levels, since we included graduated specialists from one public University in Medellín. Further research should consider other orthodontists from other universities in Colombia, in order to make regional comparisons and offer a general view of the factors affecting the QOL of orthodontist in all country. 

Accepting the above limitations, this study adds to the existing literature an exploration of the factors influencing the QOL in an important group of orthodontists through a newfangled approach (mixed methods). Further research for the orthodontic community should be focused on exploring new elements that are related to the QOL in working and social spaces. For example, to explore the contextual and social determinants of some physical and mental diseases affecting the personal and labor life in orthodontists (musculoskeletal disorders, professional burnout), since the scientific evidence in Colombia about the topic is scarce. Similarly, multiplicity of factors associated with dimensions of QOL make evident the particularities of their distribution in the subgroups, its multifactoriality and the need for intersectoriality and interdisciplinarity for its attention, promotion or research. Finally, universities and scientific societies can offer professional training in order to improve quality performance of orthodontists. 

## CONCLUSION

It is evident that there are several factors associated with a greater predictive potential for QOL in the orthodontists through different domains. The features that had the greatest influence in a positive way were: permanent contract, teaching/research activities, monthly income, resting days per week and sex. Conversely, characteristics associated negatively were low social support, mental health and rent housing. This explains the multidimensional nature of QOL in this population and corroborated for the conceptual framework that permitted to identify several social and contextual factors influencing QOL from personal and professional perspectives. 
